# The Logistical Backbone of Photoreceptor Cell Function: Complementary Mechanisms of Dietary Vitamin A Receptors and Rhodopsin Transporters

**DOI:** 10.3390/ijms25084278

**Published:** 2024-04-12

**Authors:** Matthias Leung, Jeremy Steinman, Dorothy Li, Anjelynt Lor, Andrew Gruesen, Ahmed Sadah, Frederik J. van Kuijk, Sandra R. Montezuma, Altaf A. Kondkar, Rakesh Radhakrishnan, Glenn P. Lobo

**Affiliations:** 1 Department of Ophthalmology and Visual Neurosciences, University of Minnesota, Minneapolis, MN 55455, USA; leung132@umn.edu (M.L.); stei0897@umn.edu (J.S.); li002299@umn.edu (D.L.); lor00090@umn.edu (A.L.); grues020@umn.edu (A.G.); asadah@umn.edu (A.S.); vankuijk@umn.edu (F.J.v.K.); smontezu@umn.edu (S.R.M.); 2 Department of Ophthalmology, College of Medicine, King Saud University, Riyadh 12271, Saudi Arabia; akondkar@ksu.edu.sa

**Keywords:** vitamin A, RBP4, RBPR2, STRA6, MYO1C, MYO7A, photoreceptor, rhodopsin, visual function

## Abstract

In this review, we outline our current understanding of the mechanisms involved in the absorption, storage, and transport of dietary vitamin A to the eye, and the trafficking of rhodopsin protein to the photoreceptor outer segments, which encompasses the logistical backbone required for photoreceptor cell function. Two key mechanisms of this process are emphasized in this manuscript: ocular and systemic vitamin A membrane transporters, and rhodopsin transporters. Understanding the complementary mechanisms responsible for the generation and proper transport of the retinylidene protein to the photoreceptor outer segment will eventually shed light on the importance of genes encoded by these proteins, and their relationship on normal visual function and in the pathophysiology of retinal degenerative diseases.

## 1. Introduction

The processing of visual information begins in the retina with the detection of light by photoreceptor cells. For photoreceptors to detect light, two fundamental events must take place for the generation of the retinylidene protein (rhodopsin). In no particular order, all-*trans* retinol that is derived from dietary vitamin A precursors must be absorbed and transported to the eye for the generation of 11-*cis* retinaldehyde (11-*cis* retinal), which serves as the chromophore for rod and cone opsins in the photoreceptor outer segments. Likewise, GPCR opsin proteins that are synthesized in the photoreceptor inner segments must be trafficked efficiently to the photoreceptor outer segments. In the photoreceptors, 11-*cis* retinal and opsin protein combine to form the retinylidene protein, rhodopsin, which upon the absorption of light causes the *cis*-to-*trans* isomerization of a double bond within the chromophore, inducing conformational changes in opsin and the subsequent activation of the phototransduction cascade. This is ultimately converted into a nerve impulse in the optic nerve and processed in the visual cortex of the brain, collectively initiating visual function. In all of mammalian vision, including human vision, the light induced isomerization of the chromophore 11-*cis* retinal into all-*trans* retinal and subsequent induced conformational change in the G-protein coupled receptor (GPCR) opsin is the fundamental reaction of the visual cycle. It is the first step within the phototransduction cascade, which is the process by which a photon stimulus is converted into an electrical impulse interpretable by the visual cortex of the brain [1]. While the principle of vision is well established—light-induced isomerization—a great deal of complexity within visual function lies within the constant synthesis and supply of 11-*cis* retinal precursors to the retinal pigmented epithelium (RPE), proper trafficking of rhodopsin protein to the photoreceptor outer segments (OS), and the highly specialized cellular level machinery that supports their synthesis and supply [2,3]. However, an overview of these two important processes that result in the generation and proper trafficking of the retinylidene protein is lacking. In this manuscript, we provide a succinct summary of two classes of cellular components, which work together and play a critical role in the phototransduction cascade and visual function: vitamin A membrane receptors and rhodopsin transporting proteins, which in conjunction serve as the logistical backbone in the synthesis and supply of 11-*cis* retinal and transport of the retinylidene protein/rhodopsin to the photoreceptor OS, in the maintenance of visual function.

## 2. 11-*cis* Retinal, Vitamin A Metabolism, and Vitamin A Membrane Receptors

The vitamin A metabolite, 11-*cis* retinaldehyde (11-*cis* retinal), serves as the first relevant point of contact between the photon stimuli and the visual system [4]. 11-*cis* retinal is a member within a group of vitamin A metabolites collectively called retinoids [5,6]. Retinoids exist in the body in many different forms and isomers, and the biologically relevant forms of it are also more widely known as vitamin A. The retinoid 11-*cis* retinal is highly sensitive to light and readily undergoes photoisomerization into all-*trans* retinal upon photon absorption [7]. This critical property of 11-*cis* retinal, when it is covalently bound to opsin protein as a Schiff base, allows for the detection of light stimuli, by proxy through this physical change [8].

**Figure 1:**
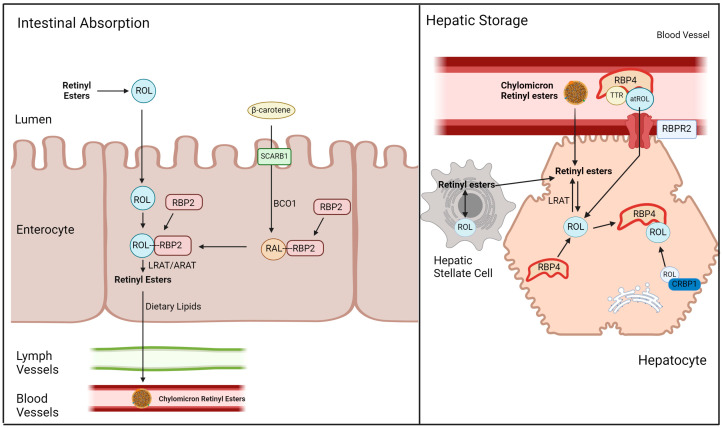
Overview of vitamin A absorption and storage. Dietary vitamin A, either in the form of retinyl esters or β-carotene, is absorbed by the intestinal enterocyte. Retinyl esters, when reduced into retinol by extracellular esterases, readily diffuses into the enterocyte. Conversely, β-carotene enters the enterocyte through the SCARB1 transporter, which is then cleaved into two molecules of all-*trans* retinal by BCO1, and then reduced into all-*trans* retinol in the cytosol. All-*trans* retinol from both sources associate with RBP2, is converted inro retinyl esters by LRAT, and released into the bloodstream in chylomicrons. A total of 70% of chylomicron bound retinyl esters are absorbed by the liver for storage. Once inside the hepatocyte, retinyl esters are converted back to all-*trans* retinol by LRAT, which can then enter the hepatic stellate cell for storage. Otherwise, all-*trans* retinol can be released into circulation when it is bound to RBP4 and TTR (holo-RBP4). Holo-RBP4 in the bloodstream can be transported back into the hepatocyte through the action of RBPR2. ROL, all-*trans* retinol; RBPR2, retinol binding protein 4 receptor 2; LRAT, lecithin retinol acyltransferase; SCARB1, scavenger receptor binding protein 1; RE, retinyl esters; RAL, all-*trans* retinal; RBP2, cellular retinol binding protein 2; TTR, transthyretin; atROL, all-*trans* retinol; RBP4, retinol binding protein 4; β-carotene monooxygenase 1, BCO1.

In the canonical understanding of vitamin A metabolism in mammalians including humans, β-carotene from plant sources and retinyl palmitate from animal sources perhaps serves as the most representative and predominant forms of dietary vitamin A (Figure 1). Dietary β-carotene is absorbed into intestinal epithelial cells through the scavenger receptor class B, member 1 (SCARB1), whereupon β-carotene oxygenase 1 (BCO1/BCMO1), a carotenoid cleaving enzyme, cleaves β-carotene into two all-*trans* retinal molecules [7,9,10]. This all-*trans* retinal binds to retinaldehyde binding protein 2 (RBP2), which is then reduced into all-*trans* retinol within the cytosol by retinol dehydrogenases (RDH). Conversely, vitamin A derived from dietary retinyl esters (RE) is hydrolyzed extracellularly into all-*trans* retinol by carboxylesterase 1 (CES1), which then diffuses into the cell. All-*trans* retinol from both retinyl palmitate and β-carotene can be found bound to RBP2 at this point in metabolism. RBP2-bound retinol cannot undergo dehydrogenation or esterification efficiently, effectively making the uptake of retinol irreversible [11,12,13,14]. The retinol-RBP2 complex interacts with lecithin retinol acyltransferase (LRAT), converting the retinol component into RE which disassociates from RBP2. This conversion also requires lecithin as a co-substrate. Retinyl esters are then packaged into nascent chylomicrons along with dietary lipids, which are secreted into the lymphatic system. Around 70% of these chylomicrons will be absorbed by the liver, while the remaining RE containing chylomicrons will be absorbed by peripheral organs (Figure 1) [7,11,12,13].

The liver can be seen as the primary control hub for whole body vitamin A homeostasis, given its capability to store vitamin A and secrete vitamin A in a form usable by peripheral organs, including the eye. The RE absorbed by the liver are first hydrolyzed into all-*trans* retinol, which then enters the hepatic stellate cell and are transformed into storage capable retinyl esters by LRAT. Stored RE is first hydrolyzed into all-*trans* retinol, which is then excreted [15,16,17,18]. All-*trans* retinol then associates with retinol binding protein 4 (RBP4) synthesized in the liver and transthyretin (TTR) to form a complex that allows movement through lymphatic and cardiovascular vessels. All-*trans* retinol bound to RBP4, which will be denoted as holo-RBP4 in this manuscript, serves as the predominant form of vitamin A within the circulation, and has shown to specifically function in providing 11-*cis* retinal precursors for visual function (Figure 1) [18,19,20,21,22].

**Figure 2:**
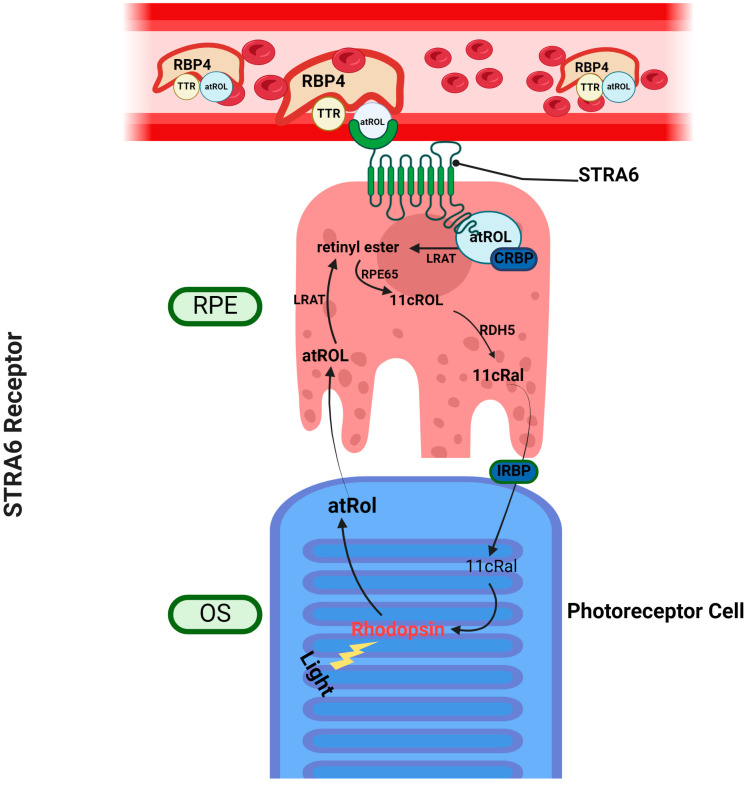
Transport of vitamin A into the eye by STRA6. Vitamin A (holo-RBP4) in the circulation is taken up by the STRA6 membrane receptor in the retinal pigment epithelium (RPE). In the RPE, all-*trans* retinol is esterified into retinyl esters by LRAT, converted into 11-*cis* retinol by RPE65, and finally converted to 11-*cis* retinal by RDH5. 11-*cis* retinal is transported into the OS, either through actions of IRBP or otherwise, and is associated with opsin to form light sensitive retinylidene protein. After phototransduction, all-*trans* retinal is converted into all-*trans* retinol by RDH8, which then is transported back into the RPE, by IRBP or otherwise, thus completing the visual cycle. RPE: retinal pigment epithelium, OS: outer segment, atROL: all-*trans* retinol, 11cRal: 11-*cis* retinal, IRBP: interphotoreceptor retinoid-binding protein, CRBP: cellular retinol-binding protein, LRAT: lecithin:retinol acyltransferase, TTR: transthyretin.

Given that holo-RBP4 is the major form of vitamin A within the circulation, as well as serving as the predominant source of vitamin A for peripheral organs including for the eye, there must be some mechanism that allows the eye to import this source of vitamin A. In 2007, an holo-RBP4 interacting receptor that is sufficiently expressed in ocular tissue was identified by Kawaguchi et al. (in the Sun Lab at UCLA), which identified the previously uncharacterized membrane protein Stimulated by Retinoic Acid 6 or Signaling Receptor and Transporter of Retinol 6 (STRA6) as a relevant receptor for RBP4 bound retinol, as well as demonstrating its ability to significantly increase intracellular vitamin A content in the presence of extracellular holo-RBP4, indicating uptake of retinol from extracellular holo-RBP4 (Figure 2) [3,23].

While visual function is maintained by the delivery of 11-*cis* retinal precursors in the form of circulatory holo-RBP4, this necessitates the maintenance of an equilibrium concentration of holo-RBP4 within the circulation. As mentioned previously, two methods of maintaining circulatory holo-RBP4 have been established: secretion of holo-RBP4 from the liver and uptake of all-*trans* retinol (ROL) from holo-RBP4 through STRA6 in ocular tissue (Figure 2). However, STRA6 is not expressed in hepatic tissue, despite the presumed role of the liver in circulatory vitamin A homeostasis, where it acts as the main site of vitamin A storage and the originator for circulatory holo-RBP4 [24,25,26,27,28,29]. Eventually, another holo-RBP4 interacting retinol receptor was discovered in 2013 by the Graham Laboratory [30]. This novel receptor, called the Retinol-Binding Protein 4 Receptor 2 (RBPR2) or STRA6-like (STRA6l), is expressed in extra-ocular tissues (predominantly in hepatic tissues), and likely serves as the mechanism by which the liver is able to intake ROL through circulatory holo-RBP4 [30,31]. With the capability to secrete and intake holo-RBP4, it is likely that the RBPR2 protein in the liver is the premier membrane protein for whole body vitamin A homeostasis (Figure 1).

## 3. The Role of Vitamin A Membrane Receptors in Sustaining Visual Function

The discovery of RBP4 facilitated retinol transport through the circulatory system as the predominant form of serum retinoid in 1968 was instrumental in the understanding of vitamin A homeostasis within biological systems [20]. Consequently, researchers began the investigation of the role of RBP4 as the circulatory chaperone of all-*trans* retinol, and the potential mechanisms in the uptake and utilization of serum holo-RBP4 by relevant cells. The existence of specific membrane receptors that can facilitate intake of retinol into specific cells have been hypothesized early on from clues derived from patterns within the physiology and biochemistry of vitamin A homeostasis. The work of John Dowling highlighted the specificity of retinol in visual homeostasis, where retinoic acid supplements given to vitamin A depleted animals were only able to remedy health and growth needs, but not visual function [5,6]. The work of Joram Heller and Dean Bok characterized the presence of holo-RBP4 binding to receptors on purified bovine RPE cells and on in vivo rat RPE cells, and indicated that these receptors were concentrated on the basal membrane of the RPE [27]. Additionally, holo-RBP4 was shown to be rapidly converted to unbound RBP4 (apo-RBP4) at the receptor, leaving RBP4 extracellularly with the radioactive-tagged retinol being transported into the cell, highlighting a key characteristic of known retinol receptors today [27]. The work of Chi-Ching Chen and Joram Heller further characterized this potential receptor by showing that the receptor is only able to interact with RBP4 bound to retinol and not free retinol, indicating the importance and specificity of RBP4 bound retinol in the intake of retinol [17].

### 3.1. Stimulated by Retinoic Acid 6—STRA6

As mentioned above, the function of STRA6 in the retinal pigmented epithelium (RPE) is the current consensus membrane receptor for ocular all-*trans* retinol intake into the eye, from circulatory holo-RBP4 (Figure 2). In the initial report by Kawaguchi et al. in *Science*, COS-1 cells transfected with bovine *Stra6* demonstrated the ability to bind to RBP4 with high affinity, as well as demonstrating the ability to significantly increase the concentration of intracellular radioactively tagged retinol (^3^[H]ROL) in the presence of extracellular holo-RBP4, indicating uptake of retinol from extracellular holo-RBP4. It should be mentioned that STRA6 activity was maximized through co-expression of LRAT in transfected cells, which as mentioned in the above sections, LRAT catalyzes the consequential step after retinol uptake; the conversion of intracellular retinol into stable storage retinyl esters. This highlights the existing synergy in which STRA6 has with previously known machinery within the pathway of vitamin A metabolism, indicating its importance in canonical vitamin A homeostasis overall [3].

Since its first identification in 2007, STRA6 has since then been further investigated by researchers. While STRA6 has been found to be highly expressed in cells found in blood–organ barriers, such as the choroid plexus of the brain, testis, kidney, spleen, and female reproductive tract, its importance and expression in the RPE has been well documented (Table 1) [23,24]. The RPE is a highly specialized single epithelial cell layer found in the retina. The RPE is extremely important in the facilitation of continuous visual functions, as it has a direct connection with the outer segments of cone and rod cells as the sole connection of photoreceptors to the cardiovascular system through the choroid, forming the blood–retina barrier. With such a premier connection to the important light sensing photoreceptors, the RPE is responsible for the maintenance and homeostasis of photoreceptors, facilitating processes such as nutrient supply and waste elimination [25,26,27,28,29,30,31,32]. Given the necessity of constant replenishment of vitamin A as the chromophore for proper visual function, and the abundant expression of STRA6 in the RPE, it has been well established that STRA6 is the vitamin A receptor responsible for the transportation of vitamin A into the outer segment of photoreceptors through the RPE.

The importance of STRA6 in the maintenance of visual function can perhaps be best demonstrated through its dysfunction. One such disease involving dysfunction on STRA6 is Matthew-Wood Syndrome, a disease characterized by a spectrum of congenital ocular deformations such as microphthalmia, congenital organ malformations, and mental developmental deficiencies. In a research sample of fetuses with Matthew-Wood Syndrome, a range of *STRA6* insertion and deletion mutations were found in transcripts [25,26,29]. The first indications of pathology regarding specific mutations in *STRA6* was found in a genetic study of a family of Irish travelers with colobomatous microphthalmia (MCOPCB), a double-nucleotide polymorphism resulting in a missense mutation (G304K) within a critical sequence of *STRA6* was found in all patients with MCOPCB [25]. In the same study, mutant *STRA6*- G304K was expressed in COS-1 cells, and these cells displayed insignificant levels of holo-RBP4 uptake when compared to cells expressing wild type STRA6 [25].

### 3.2. Retinol Binding Protein 4 Receptor 2—RBPR2

While the uptake of circulatory holo-RBP4 by peripheral tissues such as the eye was now understood through the characterization of STRA6, the absence of STRA6 expression within the liver and the lack of any alternative mechanisms for holo-RBP4 uptake in the liver and other non-ocular tissues in mice and zebrafish suggests yet an incomplete understanding of vitamin A homeostasis (Table 1) [33]. It therefore stands to reason that the liver, being the principle site of vitamin A storage as well as the site of expression for RBP4, should have the capability to interact with serum holo-RBP4 through uptake of holo-RBP4 [20]. Although the mechanism was yet unknown, this line of reasoning is supported through the observation of the interaction and affinity between the liver and serum holo-RBP4, which has previously been well established. Previous kinetic studies have shown that the liver continuously facilitates the movement of ROL associated with holo-RBP4 in and out of hepatocytes [21]. The majority of studies that worked with this interaction did so through radioactive labeling of either RBP4 or retinol itself. These studies demonstrate that the liver expresses affinity for serum holo-RBP4 [18,33], appears to facilitate the uptake of ROL into the liver from serum holo-RBP4 [19,34], and that RBP4 is not taken into the liver [22]. All of these studies indicate the existence of a second holo-RBP4 receptor with similar function and properties to STRA6, and yet is not STRA6 given that it is not expressed in hepatic tissues [23,24].

Eventually, such a receptor was discovered that fits the description as suggested by these studies. The Retinol-Binding Protein 4 Receptor 2, or RBPR2, was first characterized by Alapatt et al. within the Graham Laboratory in 2013 [30]. With the assumption that such a receptor might share structural similarity to STRA6, they performed a phylogenetic based search, which led to the discovery of mouse gene *1300002K09Rik*. The product of this gene, RBPR2, displays several structural similarities to STRA6, both have similar molecular masses of 70.1 kDa, the presence of 9–11 transmembrane domains, as well as an intracellularly soluble C-terminal domain containing 74–75 amino acids [30]. Additionally, an amino acid sequence alignment was also conducted to compare the novel RBPR2 to STRA6. This alignment study revealed six conserved amino acids, where five of the six corresponded to amino acids with known missense mutations resulting in loss of function in STRA6, suggesting homology between STRA6 and RBPR2 within these critical amino acids [30,35,36]. Physiologically and functionally, this novel receptor displayed even greater significance with regards to its hypothesized characteristics and functions. Analysis of RBPR2 mRNA expression patterns in mice determined its expression within the small intestine, spleen, adipose tissue, and most critically, in the liver (Table 1). Functionally, RBPR2 was observed to be able to not only bind to RBP4, but also able to uptake ROL from holo-RBP4. This was demonstrated through an experiment similar to the original STRA6 experiment, where ^3^[H] ROL uptake was observed in cells expressing RBPR2, and uptake was increased in cells expressing LRAT along with RBPR2 [30,31]. Another area of interest in the novel RBPR2 receptor, which concerns STRA6 as well, is the characterization of the RBP4 binding domain of RBPR2. In the amino acid sequence alignment mentioned previously conducted by Alapatt et al., a conserved sequence was proposed to be the RBP4 binding domain. This sequence contains serine, tyrosine, and leucine residues (SYL), and was found to be conserved in STRA6 and RBPR2 for both human and mice variants [30]. Through in silico analysis from our laboratory, these three residues have predicted interactions with RBP4 [35,36,37]. Mutation of these three conserved residues has also been shown to disrupt the capability of RBPR2 to perform its function, negatively affecting the capability of RBPR2 to uptake ^3^[H] holo-RBP4 in cell lines expressing mutated RBPR2, and causing significant retinal phenotypes in zebrafish with mutations in these residues [35,36,37].

While the role of STRA6 in the maintenance of visual function is apparent; by delivering ROL from serum holo-RBP4 into the visual system through the RPE, the hypothesized role of the novel RBPR2 receptor is in the facilitation of serum vitamin A homeostasis through its function in hepatic tissue [16,28,35,36]. Research regarding the importance of RBPR2 as a systemic supporter of visual function is currently limited to animal models such as mice and zebrafish, and generally includes loss of function studies, with the subsequent investigation of phenotypic effects. In zebrafish, the larval stage of development is characterized by an extremely rapid development of ocular tissue, where the visual system is fully functional by 5.5 days post fertilization (dpf) and is subsisted by vitamin A from the yolk. This allows for zebrafish larvae to be an excellent model organism for observation of retinal phenotypes. A transcription activator-like effector nuclease (TALEN) induced nonsense mutation in the zebrafish *rbpr2* gene caused severe ocular deformities in 5.5 dpf larvae, including microphthalmia, disrupted retinal layers, and cone-rod atrophy [36,37,40]. Our work on *Rbpr2* knockout (*Rbpr2*-KO) mice generated through Cre-Lox recombination has indeed demonstrated the ocular consequences of RBPR2, despite its role as a systemic receptor. When supplied with a vitamin A deficient (VAD) diet, *Rbpr2*-KO displayed significantly degraded photoreceptor layers as observed through optical coherence tomography (OCT), blood vessel leakage as observed through fluorescence angiography, decreased ocular retinoid concentrations as observed through high performance liquid chromatography (HPLC), decreased rhodopsin and cone opsin expression, and decreased electroretinogram (ERG) responses when compared to wild-type (WT) controls. However, in *Rbpr2*-KO mice supplied with a vitamin A sufficient (VAS) diet, retinal morphology defects such as degraded photoreceptor layers and blood vessel leakage were not observed. This indicates that in VAS conditions, the systemic supply of vitamin A can be maintained through alternative means such as through dietary chylomicrons, despite the lack of RBPR2. Even more critically, these experiments have demonstrated that despite the role of RBPR2 as a systemic focused vitamin A receptor, it has been proven to be highly crucial in the facilitation of visual function [31,35].

## 4. Opsin, Photoreceptor Morphology, Dynein and Kinesin Motor Proteins, and Unconventional Myosin Motor Proteins

**Figure 3:**
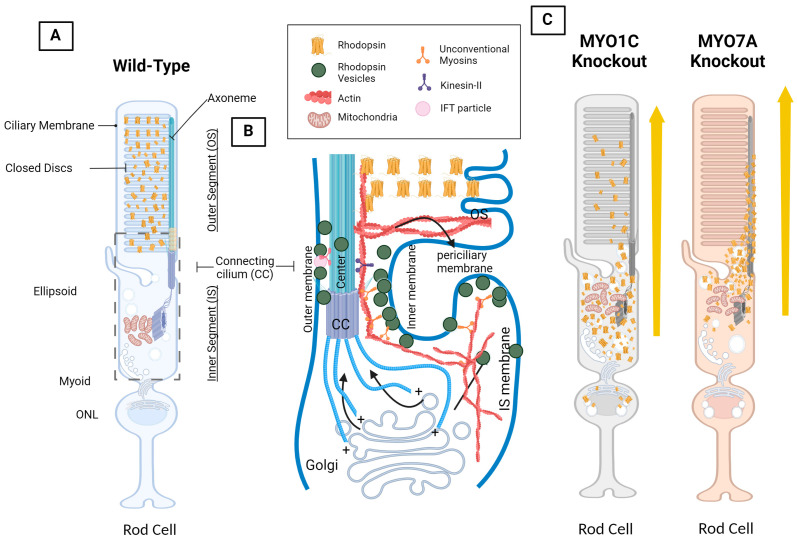
Photoreceptor structure, intraflagellar transport (IFT), and rhodopsin mistrafficking as the result of MYO1C and MYO7A inhibition in mice. (**A**) Wild-type model showing the effects of rhodopsin present in the outer segments (OS). (**B**) A model of IFT in the rod cell that occurs in all cilia and flagella. This model was created based on an immunoEM image of rhodopsin within the mouse photoreceptor [45]. Flagellar membrane proteins are carried by vesicles from the Golgi apparatus and are shown to be associated with the flagellar shaft by IFT particles on their way to the OS. When IFT particles reach the base of the OS, membrane discs are formed from membrane proteins. Black arrows demonstrate the flow of IFT through the flagellar pore complex and microtubule of the connecting cilium (CC). The CC consists of the basal body (BB), IFT particle, dynein, and kinesin II. (**C**) Model showing the adverse effects of *Myo1c* and *Myo7a* deletion in mice on rhodopsin trafficking from the photoreceptor IS to OS. In these genetic models’ rhodopsin was found mislocalized. BioRender^®^ 2.0 was used in the creation of this diagram.

The specialized cell in which vision occurs is the photoreceptor, which consists of rods responsible for scotopic vision and cones responsible for photopic and color vision (Figure 3) [41]. The photoreceptor is a highly specialized tissue and is separated into distinct compartments, reflecting the division of function within those compartments. Both cones and rods are separated into four distinct cellular compartments: the outer segment (OS), which houses an array of membranous discs embedded, with the aforementioned light sensitive 11-*cis* retinal and opsin and is the site of the visual cycle and phototransduction cascade. The other compartment is the inner segment (IS), which houses the organelles and structures found in a typical cell such as free ribosomes and the Golgi apparatus. While soluble proteins can be synthesized these free ribosomes found within the IS, transmembrane proteins such as rhodopsin are synthesized by the bound ribosomes found at the rough endoplasmic reticulum, which surrounds the nucleus within the outer nuclear layer (ONL) [42]. The connecting cilium (CC) forms a physical bridge between the IS and OS. Given that proteins such as opsins are synthesized in the IS, and that opsins must be situated in the OS to perform its function, a robust vesicular transport system consisting of motor proteins and associated filament tracks must exist, such that proteins synthesized in the IS are efficiently transported into the OS. Given the nature of photoreceptors as a primary cilium, microtubule mediated intraflagellar transport (IFT) through kinesin and dynein motor proteins has been studied extensively and has been implicated for its role in the correct transport of proteins including opsins from the IS, through the CC, and finally into the OS. Defects in IFT related proteins, such as in IFT-A and IFT-B, cause photoreceptor defects, as well as opsin accumulation within the IS of affected photoreceptors (Figure 3) [43,44].

Another group of motor proteins implicated in cellular vesicle transport is the actin mediated ATP-dependent unconventional myosin proteins. While their role is less known and apparent when compared to ciliary based protein trafficking, unconventional myosin motor proteins nonetheless are implicated to serve important roles in protein localization and transport for photoreceptor function due to its association with ocular diseases such as Usher Syndrome (USH) and Retinitis Pigmentosa (RP). While there are a multitude of myosin families and isoforms in the typical mammalian body system, with many still yet to be studied, two myosin motor proteins that are currently being studied for their role in ocular function in particular exist and are implicated in proper opsin localization: myosin motor protein 7A (MYO7A) and myosin motor protein 1C (MYO1C) (Table 2 and Figure 3) [2].

### 4.1. The VxPx Domain and the Arf4 Complex: Post-Golgi Rhodopsin Transport through a Ciliary Targeting Complex

After synthesis within the rough endoplasmic reticulum at the photoreceptor IS and post-translational modification within the Golgi, the first step is the correct transport of vesicles containing synthesized opsins towards the connecting cilium. As of the writing of this manuscript, one of the most characterized models of understanding for rhodopsin transport is the model proposed by the Deretic Laboratory from the University of New Mexico. Their research began with the observation that peptide sequences proximal to the carboxyl terminus have been shown to facilitate correct trafficking rhodopsin within photoreceptors. Earlier studies that demonstrate the importance of the carboxyl terminus involves the Q344ter mouse model, which contains a nonsense mutation resulting in truncation of the carboxyl terminus [46,47,48]. These mice display catalytically normal rhodopsin but exhibit significant mislocalization within the mouse model. Eventually, through the study of extracted retinas Southern leopard frogs, *Rana berlandieri*, the Deretic lab identified the VxPx domain. The VxPx domain is a peptide sequence that was also found in S-opsins and is found within the previously mentioned truncated region to be a sequence motif that is critical for proper localization of rhodopsin [49,50]. In particular, the VxPx domain was found to specifically with ADP-ribosylation factor 4 (ARF4), a small GTPase. ARF4, when working in conjunction with an Arf GTPase-activating protein (GAP) called ASAP1, allows for the formation of a “scaffold” that incorporates the Rab8 guanine nucleotide exchange factor (Rabin8), which in turn recruits Rab8 and Rab11, two other GTPases, and the Rab11 family-interacting protein (FIP3). The resulting complex from all of these interactions will then direct the movement of rhodopsin towards the CC from the trans-Golgi network through the photoreceptor ellipsoid region [51]. Rab11 and its effector, FIP3, have been found to form direct interactions with both dynein light intermediate chain 1 (DLIC-1), as well as dynein light intermediate chain 2 (DLIC-2); two subunits within cytoplasmic dynein-1 [52,53]. It should be noted that this interaction was demonstrated in A431 epidermal carcinoma cells, which might demonstrate different interaction mechanisms when compared to vertebrate photoreceptors. However, a study investigating the effects of cytoplasmic dynein 1 in photoreceptors has demonstrated disruptions in Rab11 based vesicle transport in mice photoreceptors, alongside disruption in photoreceptor ciliogenesis [54]. This possibly supports a model where the Arf4 ciliary targeting complex is able to interface with microtubule-based transport and is trafficked to the connecting cilium through cytoplasmic dynein 1. However, it is also possible that cytoplasmic dynein 1 is able to directly interface with rhodopsin [55]. This will be expanded in the following section.

It should also be noted that this model of understanding the molecular processes of rhodopsin remains highly contentious. Evidence against this model of understanding originate from inconsistent results from different investigators, with differing results attributed to differing methods of genetic modification in model organisms, as well as the use of different model organisms. Attempting to replicate the results from Deretic lab with an in vivo mouse model, Pearring et al. generated tamoxifen induced Arf4 floxed knockout mice (*Arf4*-KO). Upon induction of Cre recombinase with tamoxifen, these mice displayed a significant adipocyte infiltration within the exocrine pancreas, disrupted acinar cell activity, as well as a significant reduction in pancreas size overall. Notably, ciliopathic effects, such as polycystic kidney disease and retinal degeneration were not observed in these *Arf4*-KO mice [56]. Moreover, Ying et al. attempted to investigate the role of the two previously mentioned small GTPases, Rab8 and Rab11, through single knockout and double knockout in vivo mouse models. This group of researchers observed no rhodopsin mislocalization within these mice, and no notable phenotype changes when compared with the wild type controls [57]. Evidently this VxPx/Arf4 model does not yet provide a comprehensive understanding of the post-Golgi movement of rhodopsin towards the connecting cilium. The Deretic lab has since provided explanations to these discrepancies, with the most pressing one being the differences in representative animal models. The frog photoreceptor produces significantly greater molecules of rhodopsin, and contains ~6 × 10^4^ rhodopsin molecules compared to the ~2000 molecules found in the murine photoreceptor [58]. While the Deretic lab originally chose the frog as a representative animal for this exact reason, this physiological difference nonetheless most likely contributes to the differences between the mice and frog models [49]. They also attributed this difference to the method of genetic modification, where the Deretic lab utilized a dominant negative frog mutant, compared to the gene knock out mouse models seen in those other studies [59].

### 4.2. Cytoplasmic Dynein Motor Proteins and Tctex-1: Post-Golgi Rhodopsin Transport through Retrograde Microtubule Transport

In all ciliated cells, the basal body (BB), consisting of mother and daughter centrioles, serves as the microtubule organizing center (MTOC). The photoreceptor is no exception, and its basal body serves as the originator for photoreceptor microtubule structure. Within the photoreceptor, the BB is anchored within the cytoplasm, and forms the base of the axoneme. The axoneme protrudes towards the OS and forms the connecting cilium. From the BB, microtubules sprout radially throughout the cell, with the minus-end oriented towards the BB, while the plus-end is oriented radially [60,61]. Therefore, microtubule-based transport from the Golgi to the connecting cilium would conceivably involve dynein motor proteins, which traverses the microtubule toward the minus-end through retrograde movement. Evidence indicating a dynein facilitated transport at this stage is supported by the observation of in vitro interactions between the rhodopsin carboxyl terminus with t complex testis expressed 1 (Tctex-1), a subunit within cytoplasmic dynein [55]. In this study, fluorescently tagged vesicles containing rhodopsin were observed to be moving along microtubules in a dynein-based motility assay. Additionally, Tctex-1 was found to colocalize with rhodopsin in histological sections of mice photoreceptors. Empirically in vivo, mutations within cytoplasmic dynein have demonstrated rhodopsin mislocalization in zebrafish photoreceptors, as well as reduced electroretinogram responses in zebrafish [62,63]. For in vivo mice photoreceptors, Dahl et al. utilized a mice model with truncation of the motor domains and microtubule-binding domains within the cytoplasmic dynein 1 dynein heavy chain (DYNC1H1). These mice exhibited significant disruptions in the photoreceptor outer nuclear layer and inner nuclear layer, and complete absence of the outer segment. Moreover, rhodopsin trafficking is significantly disrupted, with rhodopsin congregating around the RER surrounding nucleus [64]. In another study, Kong et al. also utilized a mice model with disrupted cytoplasmic dynein 1, through deletion of the dynein light intermediate chain 1 (DLIC1). These mice displayed retinal degeneration, formation of the OS, disruption of photoreceptor ciliogenesis, as well as the previously mentioned disruption of Rab11 based vesicle transport [54].

### 4.3. Kinesin Motor Proteins and IFT: Through the Connecting Cilium to the Outer Segment

While both anterograde and retrograde IFT transport are important to the maintenance of the cilia overall, anterograde IFT transport towards the distal end was hypothesized to facilitate rhodopsin transport towards the discs in the OS. The kinesin motor protein that is currently the most studied for photoreceptor anterograde IFT transport is from the kinesin-2 family, the heterotrimeric KIF3 kinesin-2 (Table 2).

As mentioned above, the photoreceptor is a non-motile or primary cilium, and its axoneme also originates from the previously described BB and extends through the CC into the OS. Identical to centrioles, the BB is organized with the 9+0 structure with nine peripheral microtubule triplets, consisting of A-tubules, B-tubules, and C-tubules. The axoneme that protrudes from the BB is separated from the BB by a transition zone, and will only consist of A-tubules and B-tubules [65]. Like other primary cilia, the axoneme of the photoreceptor is organized in a 9+0 structure rather than the 9+2 structure found in motile cilia, with nine peripheral microtubule doublets, but not the center microtubule singlets [66]. Cilia in general requires constant maintenance, and facilitation of ciliogenesis necessitates a constant supply of materials that must be transported with a robust transport system, the photoreceptor primary cilium is no exception to this. Most notably, IFT is implicated in the maintaining tubulin supply in the dynamic process of ciliogenesis in primary cilia [67,68,69,70]. In addition to the maintenance of the microtubule axoneme itself, photoreceptor OS maintenance is facilitated by a process known as disc shedding, where accumulated waste material is constantly phagocytosed by the RPE, and disc morphogenesis is constantly occurring, requiring the mentioned robust supply of materials [71]. In most ciliated cells, including photoreceptors, IFT is the system that is most understood and is generally implicated in serving this function. However, the exact molecular mechanisms surrounding the various movements surrounding IFT trains, IFT complexes and associated motor proteins are not yet well understood. IFT is powered by both kinesin and dynein motor proteins and is structured by the microtubule based axoneme. In anterograde transport, heterotrimeric kinesin-2, associated with IFT particle B (IFT-B), are transported away from the BB on microtubules from the minus-end to the plus-end. Evidence supporting the association of IFT-B and the heterotrimeric kinesin-2 has been shown in a couple of studies. Through a yeast two-hybrid assay, Baker et al. discovered that the kinesin-2 subunit KIF3B was shown to form an interaction with IFT20, a subunit within IFT-B. Liang et al. investigated the role of phosphorylation of heterotrimeric kinesin-2 by the calcium-dependent protein kinase CrCDPK1 in *Chlamydomonas*, where this phosphorylation event has been shown to disrupt the interaction between kinesin-2 and its cargo [72]. Liang et al. expanded upon this study and discovered that phosphorylation of kinesin-2 disrupts its interaction with IFT-B in particular [73]. The heterotrimeric KIF3 kinesin-2 consists of the motor subunits KIF3A, KIF3B, and the KAP3 regulatory subunit [74]. Within this kinesin complex, KIF3B has been shown to interact with the IFT-B-connecting tetramer, consisting of IFT38, IFT52, IFT57, IFT88 [75].

Once the IFT complex has been transported to the distal end of the axoneme, the IFT cargo is unloaded, the remaining IFT complex is remodeled, heterotrimeric kinesin-2 is inactivated and dissociates, and the retrograde motor is activated and assumes function [76]. Dynein motor proteins, specifically cytoplasmic dynein-2, which were thought to be associated with IFT particle A (IFT-A) [76,77]. Since IFT-A and IFT-B are part of the same complex, cytoplasmic dynein-2 are therefore transported by heterotrimeric kinesin-2 to the distal end of the axoneme as its cargo. Once at the distal end, cytoplasmic dynein-2 assume the role as the functioning retrograde motor protein and transport IFT-A and IFT-B towards the BB from the plus-end to the minus-end through retrograde transport [78,79,80]. Rosenbaum and Witman have compiled an excellent and comprehensive review on IFT as a whole [81].

Empirically, mice with conditional rod *Kif3a* knocked out (*Kif3a*-KO) with Cre-Lox recombination demonstrated opsin accumulation within the IS and CC, leading to eventual photoreceptor death. Notably, these researchers concluded that *Kif3a*-KO mice demonstrated no affect to CC integrity, and that apoptosis of the photoreceptor is occurs as a result of abnormal opsin localization [43]. Again utilizing a Cre-Lox recombination to generate a mouse model of a conditional rod *Kif3a*-KO model, these mice demonstrated similar results with opsin mislocalization and eventual apoptosis, but also observed arrestin accumulation [44]. However, it should be noted that IFT presents a yet incomplete understanding of opsin transport, because conflicting results about the effects of KIF3 complex disruption exist for different studies. In another more recent study, conditional tamoxifen induced Cre expression in mice with floxed *Kif3a* apparently expressed displayed normal trafficking of rhodopsin and cone opsin. Photoreceptor degeneration and eventual cell death was still observed by this group however, which the researchers have attributed to disrupted axoneme maintenance [82]. Furthermore, these same researchers expanded their study to include a *Kif17* knock out *Kif17*-KO mice model, another member of the kinesin-2 family that is also attributed to function in IFT. *Kif17*-KO mice and *Kif3* and *Kif17* double knockout mice did not display rhodopsin mis-trafficking [83].

Overall, while IFT presents as a promising system for the understanding of opsin trafficking through the CC, this explanation is not airtight. Other explanations, such as in unexplored redundant systems or perhaps other motor proteins, need to be further investigated to truly understand this critical aspect of visual function.

### 4.4. Myosin Motor Protein 7A: MYO7A

MYO7A has previously been designated as the cause of a known visual and auditory disease called Usher Syndrome 1B (USH1B). USH1B is an autosomal recessive disease characterized by the dual dysfunction of cochlear and retinal tissue, resulting in symptoms such as loss of hearing, loss of equilibrioception, and loss of visual function through Retinitis Pigmentosa (RP) [84,85]. MYO7A is also an unconventional myosin protein that was also found to be expressed in human photoreceptors, with a particularly high concentration in the connecting cilium as well as in the RPE [86,87]. MYO7A has been implicated to facilitate the transport of rhodopsin from the IS to the OS through the connecting cilium, which is possible given its high expression in the connecting cilium (Figure 3) [2,86]. Several roles for MYO7A within the connecting cilium have been implicated in the past, including the transport of proteins to the outer segment for photoreceptor disc assembly, and also for the transport of opsin synthesized in the IS to the OS (Table 2) [86,88].

Within the RPE layer, MYO7A was observed to support the function of retinoid isomerohydrolase (RPE65), an enzyme that catalyzes a critical reaction within the visual cycle, the conversion of all-*trans* retinyl ester into 11-*cis* retinol, allowing for the regeneration of 11-*cis* retinal for photo-isomerization. MYO7A was found to co-immunoprecipitate with RPE65, and facilitates the light dependent translocation of RPE65 to the smooth endoplasmic reticulum within the RPE, which lowers its rate of proteolysis [89]. Another role for MYO7A within the RPE is its participation in melanosome transport around the RPE, which help scatter light and protect the retina from photooxidation [90,91]. Given all these functions, as well as its proven association with a known disease with an ocular phenotype, MYO7A presents as a multifaceted component in the facilitation of visual function.

### 4.5. Unconventional Myosin Motor Protein 1C, MYO1C: A New Player in Understanding Rod Opsin Transport in Visual Function

MYO1C, another unconventional myosin motor protein like MYO7A, presents as a novel candidate in the further understanding of opsin trafficking in visual function, with new empirical data indicating disruption of visual function in knockout mouse models. Through co-immunoprecipitation assays of mice retinal lysate between WT and *Myo1c*-knockout (*Myo1c*-KO) mice, rhodopsin was observed to co-immunoprecipitate with MYO1C, and thus demonstrated a direct interaction between rhodopsin and MYO1C, indicating rhodopsin as a possible cargo for MYO1C [92]. Once the interaction between MYO1C and rhodopsin was confirmed, the role of MYO1C was further explored using a *Myo1c*-KO mouse line. ERG testing in the *Myo1c* -KO models exhibited considerable *b*-wave deficiencies, and by the six-month time point, *Myo1c*-KO mice demonstrated *a*-wave amplitude deficits in addition to progressive *b*-wave loss of function. As predicted, immunofluorescent imaging of two-month *Myo1c*-KO retina sections demonstrated defective rhodopsin trafficking to the photoreceptor OS. Six-month *Myo1c*-KO retinal sections demonstrated an expected progressively severe mistrafficking of rhodopsin, with a large quantity of rhodopsin found in the IS and cell body. Interestingly, the two and six month sections showed progressively misshapen OS cones, suggesting a structural responsibility of MYO1C in the OS [2,92]. Furthermore, in silico studies have revealed that the VxPx domain of rhodopsin may participate in the non-covalent binding of rhodopsin to MYO1C [2,92]. This is the same peptide sequence described above that was observed function as a CC or OS directing signal through its association with the Arf4 complex and cytoplasmic dynein. It is conceivable that this observation could indicate MYO1C as a redundant motor protein pathway, and could work alongside cytoplasmic dynein and kinesin motor proteins in IFT to facilitate opsin transport, since IFT does not completely explain opsin transport [82,83]. More work will need to be done to completely understand the potential role of MYO1C in opsin trafficking, including potential complex formations, actin infrastructure in photoreceptors, or perhaps alternate explanations for the phenotypes observed in *Myo1c*-KO mouse models [93,94]. Given the nature of MYO1C as an actin-based motor protein, rhodopsin mistrafficking resulting from *Myo1c* knockout in model organisms, as well as the in silico prediction of MYO1C/rhodopsin interactions, current research indicates MYO1C as a novel candidate in the yet fully understood topic of opsin trafficking.

## 5. The Visual Cycle and Phototransduction Cascade—Actions of Vitamin A Receptors and Myosin Motor Proteins Converge and Form the Retinylidene Protein

**Figure 4:**
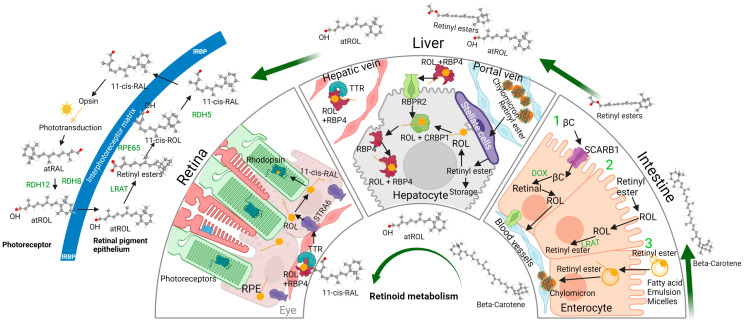
Logistical role for vitamin A receptors and motor proteins in visual function. For the human eye to detect light, two events must take place in the photoreceptors for the generation of the retinylidene protein. Dietary vitamin A precursors must be absorbed and transported to the eye for the generation of 11-*cis* retinal, which serves as the chromophore for rod and cone opsins in the photoreceptor outer segments. Herein, two vitamin A receptors, retinol binding protein 4 receptor 2 (RBPR2), and stimulated by retinoic acid 4 (STRA6) receptors, fulfil this role. Likewise, GPCR opsin proteins that are synthesized in the photoreceptor inner segments must be trafficked to the photoreceptor outer segments. Herein, unconventional motor proteins (MYO1C, MYO7A), among dynein’s, and kinesins, have been shown to be involved in rhodopsin trafficking. In the photoreceptor outer segments, the retinylidene protein, which upon the absorption of light causes the *cis*-to-*trans* isomerization of a double bond within the chromophore, inducing conformational changes in opsin and subsequently activation of the phototransduction cascade of events.

All-*trans* retinol associated with RBP4 is the fundamental serum transport form of vitamin A and is the form of vitamin A that serves as the precursor for the visual chromophore 11-*cis* retinal. Holo-RBP4 is secreted from the liver into the serum to reach the systemic tissues that require vitamin A for its function. Concurrently, holo-RBP4 is also reabsorbed into the liver through the action of RBPR2 as a means of regulating systemic vitamin A homeostasis, which in turn allows for efficient uptake of ROL into ocular tissue for visual function [30,37]. ROL from serum holo-RBP4 is then provided entry into the RPE through STRA6, the critical receptor that uptakes ROL from holo-RBP4 while leaving RBP4 extracellularly in the serum [3]. Within the RPE, ROL undergoes the visual cycle, a process by which 11-*cis* retinal is synthesized from ROL, and where 11-*cis* retinal that is photoisomerized into all-*trans* retinal can be reconverted back into ROL, thus completing the visual cycle. ROL that enters the RPE cell from STRA6 is first esterified into all-*trans* retinyl ester by LRAT, which is then converted into 11-*cis* retinol by RPE65. As mentioned above, RPE65 is dependent on the actions of MYO7A, an unconventional myosin protein, for proper translocation to the smooth endoplasmic reticulum. 11-*cis* retinol then is isomerized into 11-*cis* retinal by retinol dehydrogenase 5, which is then transported into the outer segment of the photoreceptor by interphotoreceptor retinoid-binding protein (IRBP) [7,95]. It should be noted however, that the role of IRBP within this functions is controversial, because mice models with IRBP knocked out do not exhibit disruptions in the visual cycle [96]. This concludes the process for the procurement of 11-*cis* retinal into its proper place for visual function, which is facilitated by the combined efforts of both vitamin A receptors and unconventional myosin motor proteins (Figure 4).

Opsins in the form of rhodopsin and cone opsins on the other hand, originate from the rough endoplasmic reticulum surrounding the nucleus within the ONL. For rhodopsin, through the ciliary targeting Arf4 complex or cytoplasmic dynein-1 with Tctex-1, transports rhodopsin to the base of the CC. This initiates rhodopsin transport from the inner segment to the outer segment, either through IFT or another yet unknown method. MYO7A can also potentially fulfill a similar role in the transport of opsins and photoreceptor disc proteins to the outer segment. Henceforth, the procurement of opsin into its proper place for visual function has also been completed, owing to the effects of motor proteins [50,93,94].

With both 11-*cis* retinal and opsin in their proper place within the photoreceptor discs in the outer segment, the critical process of the phototransduction cascade can finally occur (Figure 4). While separated initially, 11-*cis* retinal and opsin are covalently linked through a Schiff base on lysine residue at position 296 on rhodopsin [8]. Upon photoisomerization of 11-*cis* retinal to all-*trans* retinal with the absorption of a photon, the chromophore-opsin complex changes conformation into the activated form of the chromophore-opsin complex, known as metarhodopsin II. Given the identity of opsin as a G-protein coupled receptor, metarhodopsin II can activate the G-protein α-subunit called transducin, which then activates a phosphodiesterase known as PDE6. PDE6 then facilitates the hydrolysis of cGMP into GMP, thus lowering the concentration of cGMP, which closes the cyclic nucleotide gated (CNG) channel, closing access of sodium and calcium ions from the extracellular space [97]. The closing of the CNG channels, along with continued efflux of intracellular potassium and calcium ions from the Na^+^/Ca^2+^, K^+^ exchanger (NCKX), causes hyperpolarization of the photoreceptor, which causes the closing of calcium channels [98,99,100]. With the closing of calcium ion channels, the photoreceptor ceases to secrete the activating neurotransmitter glutamate in the photoreceptor synapse, which is then registered as a depolarization in on-center bipolar neurons, leading to continued activation of downstream ocular neurons, culminating as a signal through the optic nerve [101,102].

## 6. Concluding Remarks and Future Directions

Much progress has been made in elucidating the mechanisms of dietary vitamin A transport to the eye and in the trafficking of opsins to the photoreceptor OS, which composes the two logistical events that are critical for human visual function; the generation of the retinylidene protein that serves as the interface between the photon stimulus and phototransduction. Given its importance in visual function, it is not surprising that mutations in the genes involved in vitamin A transport or opsin trafficking are associated with known ocular diseases, such as Matthew-Wood Syndrome, Leber congenital Amaurosis, fundus albipunctatus, Usher Syndrome, or Retinitis Pigmentosa. Consequentially, the importance of the dietary vitamin A derivative 11-*cis* retinal as the chromophore for rod and cone opsins in the photoreceptors necessitates that the mechanisms that control the supply, storage, and transport of dietary vitamin A to the eye and the proper trafficking of GPCR opsins to the photoreceptor OS are investigated and well understood.

In pushing the frontier within this critical area of study within visual function, our laboratory utilizes a combination of structural modeling, in vitro biochemical analysis, and genetically modified animal models to investigate the role of both STRA6 and RBPR2 in the transport of all-*trans* ROL from RBP4 (holo-RBP4) from dietary vitamin A precursors to the eye, as well as the role of myosin motor proteins (MYO1C) in the trafficking of opsin to the photoreceptor OS. In particular, our current and in-development novel animal models allow for the study of the synergistic roles of vitamin A receptors and motor proteins in visual function and diseases states of vitamin A deprivation or defects in trafficking of opsin to the photoreceptor outer segments.

Although much has since been discovered in both vitamin A metabolism and opsin transport, there yet exists a wide frontier in which further work and progress can be made. In the realm of vitamin A metabolism, the potential signaling pathways between the liver and peripheral tissues, which might signal for the liver to release more holo-RBP4 in times of peripheral retinoid deficiency, is still unknown. Additionally, the exact three-dimensional structure of RBPR2 has yet to be demonstrated empirically through techniques such as cryogenic electron microscopy (cryo-EM). The three-dimensional structure of STRA6 was only discovered recently in 2016, and much about STRA6, such as the mechanism of ROL intake and its association with calmodulin, was only elucidated after a successful a cryo-EM characterization [103,104]. The determination of the exact three-dimensional structure of RBPR2 might yet lead to further understanding of vitamin A metabolism. In the realm of opsin transport, one potential avenue of further development is in the development of additional *Myo1c* knock-out animal models. A mouse model only currently exists for *Myo1c* deficiency, and the development of other animal models will allow for more varied in vivo studies and allow for us to take advantage of the benefits inherent in other animal model species.

## Figures and Tables

**Table 1:** Tissue expression patterns of RBPR2 and STRA6 in mouse and zebrafish. Colored sections indicate respective vitamin A receptor expression in various tissue in mice and zebrafish (black, highly expressed; grey, expressed; red, not expressed; white, not reported) that have been reported in the literature [21,31,32,33,34,35,36,37,38,39].

Mouse Expression	Retinol Binding Protein Receptor 2 (RBPR2)	Stimulated by Retinoic Acid 6 (STRA6)
RPE/Eye		
Brain		
Liver		
Intestine		
Spleen		
Kidney		
Adipose		
Lung		
**Zebrafish** **Expression**	**Retinol Binding protein Receptor 2** **(RBPR2)**	**Stimulated by Retinoic Acid 6** **(STRA6)**
RPE		
Brain		
Liver		
Intestine		
Spleen		
Kidney		
Adipose		
Pancreas		

**Table 2:** Expression patterns and proposed function of unconventional Myosin Motor Proteins and Rhodopsin transporters in vertebrates.

Protein	Type	Expression	Proposed Function
Myosin 1C	Unconventional motor protein	Widely distributed; Cytoplasmic as well as nuclear isoforms. Mouse Photoreceptors Upper tip link in the mouse ear inner hair cells	Transcription initiation; Intracellular vesicle transport to plasma membrane (in mice); may form adaptation motor complex; role in inner ear function; Opsin transport to Photoreceptor Outer Segments and Visual Function; Mouse Vitreous; Auditory function, hair cell adaption and Actin binding; Missense mutations reported in patients with sensorineural hearing loss
Myosin 7A	Unconventional motor protein	Retinal Pigmented Epithelium and Photoreceptor cilium	Rhodopsin transport; RPE65 localization in RPE
Kinesin	Motor	Photoreceptors	Microtubule-based anterograde intracellular transport
Kif3a	Motor	Photoreceptors	Rhodopsin transport
Dynein	Motor	Photoreceptors	Microtubule-based retrograde intracellular transport
